# Efficacy and safety of video laryngoscope in indwelling gastric tube in severe coma patients: A protocol for systematic review and meta-analysis

**DOI:** 10.1097/MD.0000000000032036

**Published:** 2022-12-30

**Authors:** Mengying Gao, Guoying Wang, Yunjiao Zhang, Mei Han, Huiping Niu, Wenlin Li

**Affiliations:** a Department of Emergency Medicine, The Second Hospital of Hebei Medical University, Hebei, China; b Department of Emergency Medicine, The Third People’s Hospital of Hengshui, Hebei, China; c Department of Intensive Care Medicine, The Second Hospital of Hebei Medical University, Hebei, China; d Department of Emergency Medicine, The Fourth Hospital of Hebei Medical University, Hebei, China; e Endoscopy Room, The Second Hospital of Hebei Medical University, Hebei, China.

**Keywords:** indwelling gastric tube, meta-analysis, protocol, severe coma, video laryngoscope

## Abstract

We designed this systematic review and meta-analysis protocol to provide new medical evidence for clinical management by comparing the prognostic outcomes of visual laryngoscopy with those of conventional blinded insertion methods.

**Methods:**

We will intend to search English databases including Medicine, Embase, Web of Science, Cochrane Central Register of Controlled Trials, Scopus, and Google Scholar. The Chinese databases, such as Wanfang, China Knowledge Network, and China Biomedical Literature Database will also be searched. The outcome measures include intubation success rate, pain score, intubation-related complications, patient satisfaction, operation time, and cost. The Jadad scale will be used to evaluate the methodological quality of each randomized controlled trial in this meta-analysis. We will use the Methodological Index of Non-Randomized Studies criteria to assess the risk of bias in non-randomized study. An *I*^2^ value greater than 50% indicates the presence of significant heterogeneity. *P* < .05 in a 2-tailed test is considered statistically significant.

**Results:**

It is hypothesized that video laryngoscope will provide better outcomes compared with traditional blind gastric tube insertion.

**Conclusions:**

The results of our review will be reported strictly following the PRISMA criteria and the review will add to the existing literature by showing compelling evidence and improved guidance in clinic settings.

## 1. Introduction

Comatose patients in the intensive care unit (ICU) frequently require enteral nutritional support or gastrointestinal decompression, thus indwelling gastric tube is a routine operation item in nursing work.^[1,2]^ However, gastric tube insertion is prone to reflexive swallowing reaction, resulting in difficult intubation or pharyngeal injury.^[3]^ In addition, comatose patients also have a combined endotracheal tube that compresses the esophageal opening, which further increases the difficulty of intubation.^[4]^ Therefore, how to safely and quickly insert a gastric tube is a challenge for ICU nursing staff.

Previously, blind gastric tube insertion was the main method used in clinical practice. However, its success rate of 1-time insertion was low, and repeated exploratory operation during intubation tended to aggravate the pain of patients. Previous literature has shown that repeated intubation can easily damage the mucosa of the pharynx, and in serious cases, it can accidentally enter the airway, causing choking and asphyxiation.^[5]^ In recent years, visualization has been gradually applied in clinical work. Video laryngoscope guided gastric tube indwelling method can accurately locate the esophageal orifice by means of the clear image provided by the video laryngoscope screen, and its design fully considers the airway structure of human body. This technology also increases the visual field of the operator, theoretically can effectively reduce the throat injury caused by blind insertion and improve the safety and success rate of indwelling gastric tube.^[6–8]^

Despite the great theoretical advantages of visual laryngoscopy, there is still a lack of relevant evidence to prove its effectiveness. Therefore, we designed this systematic review and meta-analysis protocol to provide new medical evidence for clinical management by comparing the prognostic outcomes of visual laryngoscopy with those of conventional blinded insertion methods. It is hypothesized that video laryngoscope will provide better outcomes compared with traditional blind gastric tube insertion.

## 2. Materials and Methods

### 2.1. Study protocol registration

The protocol was written following the preferred reporting items for systematic reviews and meta-analyses protocols statement guidelines.^[9]^ The prospective registration has been approved by the PROSPERO and the registration number is CRD42022369734. Ethical approval and patient consent are not required because this study is a literature-based study.

### 2.2. Searching strategy

We will intend to search English databases including Medicine, Embase, Web of Science, Cochrane Central Register of Controlled Trials, Scopus, and Google Scholar. Given the comprehensiveness of the systematic review, we did not impose language, time, or publication status restrictions on the search process. We will use the following search strings in the English databases: (critical coma OR serious coma OR severe coma) AND (visual laryngoscope OR visible laryngoscope OR visualization laryngoscope) AND (gastric tube OR gastrostomy tube). We use a combination of keywords and index terms to achieve the highest sensitivity. In addition, we will also conduct a blanket search of Chinese databases, such as Wanfang, China Knowledge Network, and China Biomedical Literature Database. Finally, we search the references of the target literature manually to further reduce the possibility of literature omission.

### 2.3. Eligibility criteria

The inclusion criteria for this meta-analysis were set as follows: comatose patients in the ICU; the intervention and control groups were the visual laryngoscopy and blinded gastric tube, respectively; one of the following outcome measures including intubation success rate, pain score, intubation-related complications, patient satisfaction, operation time, and cost should be provided in the text. The target articles for inclusion was not limited to randomized controlled trial. Exclusion criteria included the following: full articles with serious flaws in methodological design; no validated control group; incomplete data; conference abstracts, technical presentations, case reports, envelopes, reviews, and animal studies.

### 2.4. Data extraction

After explicit inclusion, 2 independent authors will have extracted the following descriptive raw information from the selected studies: study characteristics, such as authors, study design, study language, year of publication, and mean follow-up period; patient demographic details including sample size, mean age, body mass index, and sex ratio; and details of the intervention and cohort, and outcome measures. When studies report outcomes for multiple follow-up times, data will be collected in different subgroups for similar periods. If data are missing or can not be extracted directly, we will contact the appropriate author to ensure that the information is complete. If necessary, we will abandon the extraction of incomplete data. Data will be organized into laboratory tables and Excel spreadsheets in duplicate. All data extraction will be performed by the 2 independent authors. In the event of any discrepancies, these issues are resolved either by a third investigator or by agreement between the 2 original investigators.

### 2.5. Data analysis

The mean differences in 95% confidence intervals for continuous outcomes such as pain scores, patient satisfaction, operative time, and cost and the risk ratios in 95% confidence intervals for dichotomous outcomes including intubation success and complications will be used to estimate the combined effects in this article. All meta-analyses are performed using random effects models with Der Simonian and Laird weights. Heterogeneity is tested using the Cochran *Q* statistic (*P* < .1) and quantified using the *I*^2^ statistic, which describes the change in effect size attributable to study heterogeneity. An *I*^2^ value greater than 50% indicates the presence of significant heterogeneity. Sensitivity analyses will be performed to detect the effect of individual studies on the overall estimate by sequentially omitting a study when necessary. If a limited number of studies are included, publication bias will not be assessed. Otherwise, publication bias will be assessed using the Begg’s funnel plot. *P* < .05 in a 2-tailed test is considered statistically significant. All statistical analyses will be performed using Review Manager version 5.3 (The Cochrane Collaboration, Software Update, Oxford, UK).

### 2.6. Qualitative evaluation of cited studies

The Jadad scale will be used to evaluate the methodological quality of each randomized controlled trial in this meta-analysis.^[10]^ The scale consists of 3 evaluation elements: randomization, blinding, and dropout. One point will be assigned to each element if it is mentioned in the article, and an additional point will be assigned if the randomization and/or blinding methods are appropriately described. One point will be deducted for improper randomization and/or blinding methods, or failure to document withdrawal. Articles with a Jadad score ≤ 2 are considered to be of low quality. Studies will be considered high quality if the Jadad score is ≥ 3. We will use the methodological index of non-randomized studies criteria to assess the risk of bias in non-randomized study. The methodological index of non-randomized studies criteria is a 12-item checklist that scores various parameters to assign an overall risk of bias score out of 24 points.^[11]^

## 3. Discussion

Retained gastric tube is a channel to provide nutritional support for comatose patients in ICU, and it is a frequent operation in ICU nursing. However, comatose patients who cannot cooperate with the operation and airway intubation increase the difficulty of gastric tube insertion. Visual laryngoscopy can fully expose the pharyngeal view and prevent injury and inflammation due to repeated blind insertion. Despite the great theoretical advantages of visual laryngoscopy, there is still a lack of relevant evidence to prove its effectiveness. Therefore, we designed this systematic review and meta-analysis protocol to provide new medical evidence for clinical management by comparing the prognostic outcomes of visual laryngoscopy with those of conventional blinded insertion methods. It is hypothesized that video laryngoscope will provide better outcomes compared with traditional blind gastric tube insertion.

## Author contributions

**Conceptualization:** Mei Han, Huiping Niu.

**Data curation:** Mengying Gao, Guoying Wang.

**Formal analysis:** Mengying Gao, Guoying Wang.

**Funding acquisition:** Wenlin Li.

**Investigation:** Mengying Gao, Guoying Wang.

**Methodology:** Mei Han.

**Resources:** Wenlin Li.

**Software:** Yunjiao Zhang.

**Supervision:** Wenlin Li.

**Validation:** Huiping Niu.

**Visualization:** Huiping Niu.

**Writing – original draft:** Mengying Gao, Guoying Wang.

**Writing – review & editing:** Yunjiao Zhang.
